# The influence of HKUST-1 and MOF-76 hand grinding/mechanical activation on stability, particle size, textural properties and carbon dioxide sorption

**DOI:** 10.1038/s41598-024-66432-z

**Published:** 2024-07-04

**Authors:** Tomáš Zelenka, Matej Baláž, Marta Férová, Pavel Diko, Jozef Bednarčík, Alexandra Királyová, Ľuboš Zauška, Radovan Bureš, Pooja Sharda, Nikolas Király, Aleš Badač, Jana Vyhlídalová, Milica Želinská, Miroslav Almáši

**Affiliations:** 1 Department of Chemistry, Faculty of Science, University of Ostrava, 30. Dubna 22, 702 00 Ostrava, Czech Republic; 2 Institute of Geotechnics, Slovak Academy of Sciences, Watsonova 45, 040 01 Košice, Slovak Republic; 3 Institute of Experimental Physics, Slovak Academy of Sciences, Watsonova 47, 040 01 Košice, Slovak Republic; 4 Department of Inorganic Chemistry, Faculty of Science, P. J. Šafárik University, Moyzesova 11, 041 01 Košice, Slovak Republic; 5 Institute of Materials Research, Slovak Academy of Sciences, Watsonova 47, 040 01 Košice, Slovak Republic; 6 Department of Physics, School of Applied Science, Suresh Gyan Vihar University, Jaipur, I-302017 India

**Keywords:** Metal–organic frameworks, HKUST-1/MOF-76, Mechanical activation, Particle size, Nitrogen adsorption, Carbon dioxide storage, Chemistry, Materials science

## Abstract

In this study, we explore the mechanical treatment of two metal–organic frameworks (MOFs), HKUST-1 and MOF-76, applying various milling methods to assess their impact on stability, porosity, and CO_2_ adsorption capacity. The effects of different mechanical grinding techniques, such as high-energy ball milling and hand grinding, on these MOFs were compared. The impact of milling time, milling speed and ball size during high-energy ball milling was assessed via the Design of Experiments methodology, namely using a 3^3^ Taguchi orthogonal array. The results highlight a marked improvement in CO_2_ adsorption capacity for HKUST-1 through hand milling, increasing from an initial 25.70 wt.% (5.84 mmol g^-1^) to 41.37 wt.% (9.40 mmol g^-1^), marking a significant 38% increase. In contrast, high-energy ball milling seems to worsen this property, diminishing the CO_2_ adsorption abilities of the materials. Notably, MOF-76 shows resistance to hand grinding, closely resembling the original sample’s performance. Hand grinding also proved to be well reproducible. These findings clarify the complex effects of mechanical milling on MOF materials, emphasising the necessity of choosing the proper processing techniques to enhance their stability, texture, and performance in CO_2_ capture and storage applications.

## Introduction

Metal–organic frameworks (MOFs) represent a class of porous materials with exceptional structural diversity, tunability, porosity and surface area. MOFs consist of metal nodes or clusters interconnected by organic molecules (linkers), forming a three-dimensional framework with well-defined and regularly arranged pores^1,2^. The high degree of customisation in MOF synthesis allows for precise control over their properties, such as pore size, surface area, and functionality. MOFs have attracted significant attention across various scientific disciplines due to their remarkable properties and potential applications. Their large surface area and pore volume make them excellent candidates for gas capture, storage and separation^3,4^, including environmentally harmful gases/organic pollutants^5,6^ and the selective adsorption of specific molecules^7^. The tunable nature of MOFs enables the design of materials for applications in heterogeneous catalysis, where the pore environment and metal sites can be tailored to enhance catalytic reactivity and selectivity^8–10^. Furthermore, metal–organic frameworks have shown promise in drug delivery systems, as their porous structures can encapsulate and protect drug molecules while allowing controlled release^11–13^. Their ability to incorporate different functional groups within the framework opens up possibilities for applications in sensing, magnetism, optics, and electronics^14^.

The resulting design, dimensionality, and pore shape of MOF materials depend on appropriately chosen building components, especially the linker. Benzene-1,3,5-tricarboxylic acid (H_3_BTC) is a widely utilised organic linker in the synthesis of metal–organic frameworks (MOFs) due to its versatile coordination ability and structural features. H_3_BTC, also known as trimesic acid, consists of a benzene ring with three carboxylic acid groups located at the 1, 3, and 5 positions. The mentioned carboxylic acid has been employed in the fabrication of numerous MOF materials, including HKUST-1^15^, MIL-100^16^, MOF-76^17^, MOF-505 (NOTT-100)^18^, MOF-808^19^ and STAM-1^20,21^. These are just a few examples of the diverse range of MOFs prepared using BTC(-III) linker as a key building block^22^.

**Figure 1:**
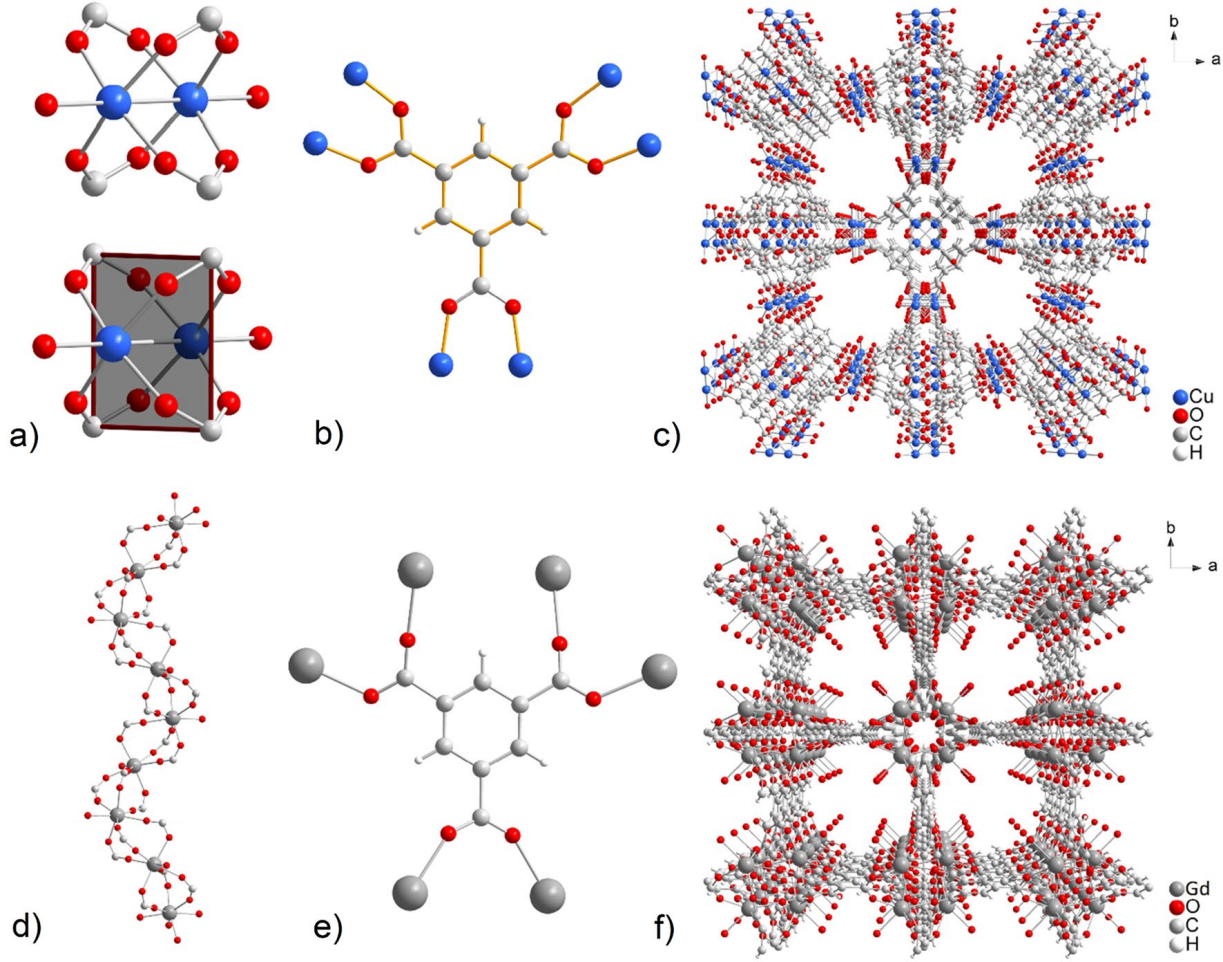
(**a**) Paddle-wheel cluster with square planar SBU present in HKUST-1, (**b**) coordination mode of BTC(-III) linker and (**c**) final framework of HKUST-1. (**d**) Helical strands present in the crystal structure of MOF-76 and (**e**) coordination fashion of BTC(-III) ligand with (**f**) a view of the final framework containing 1D channels.

HKUST-1 (also known as MOF-199 or CuBTC) with the chemical composition [Cu_3_(BTC)_2_(H_2_O)_3_] belongs to one of the first and best-known MOF materials, the synthesis of which was first published in 1999 by Chui et al.^15^. The crystal structure of HKUST-1 exhibits a cubic topology and consists of two pentacoordinate copper(II) cations, which are bridged by four carboxylate groups of four BTC(-III) molecules coordinated in a *syn-syn* mode (see Figs. 1a, b). The coordination polyhedron of Cu(II) central atoms is a tetragonal pyramid. The described carboxylate oxygens form the base, and the oxygen donor atom of the coordinated water molecule completes the top of the pyramid. The aforementioned coordination of building blocks results in a paddle-wheel cluster [Cu_2_(COO)_4_(H_2_O)_2_] with a square secondary building unit (SBU), as shown in Fig. 1(b). A mutual combination of SBUs and BTC(-III) linkers results in a microporous 3D framework containing large square-shaped pores with sizes of 9 × 9 Å^2^ (see Fig. 1c). Its high stability, modifiability, and tunability make it a promising candidate for developing efficient and selective heterocatalytic systems. The presence of Cu(II) ions with coordinatively unsaturated sites in its structure grants HKUST-1 redox activity and Lewis acidity, making it a potential catalyst or catalyst support. HKUST-1 has been explored in various catalytic reactions, including oxidation^23^, de/hydrogenation^24,25^, C–C and C–N coupling reactions^26,27^, Knoevenagel condensation^28^, Beckmann rearrangement^29^, Friedel–Crafts alkylation^30^, aziridination^31^, and others. This material is also intensively studied as a gas adsorbent, showing high capture/storage capacities for CO_2_^32^, H_2_^33,34^, CH_4_^35,36^, NO^37^, NH_3_^38^ and C_2_H_2_^39^. In drug delivery, HKUST-1 was applied as a drug carrier for paracetamol^40^, ibuprofen, anethole, guaiacol^41^ and curcumin^42^. The unique textural properties of HKUST-1 allow for the efficient loading of mentioned drugs, providing opportunities for targeted drug delivery and enhanced therapeutic outcomes. Additionally, HKUST-1 has shown potential in other applications such as sensing, optoelectronics, and energy storage. Its unique properties make it suitable for sensing gases and detecting analytes^43,44^, making it valuable in environmental monitoring and chemical sensing applications^45,46^. In the field of optoelectronics, HKUST-1's electronic and optical properties have been harnessed for applications like light-emitting devices and sensors^47,48^. Furthermore, the stability and potential for ion exchange in HKUST-1 have made it a contender for energy storage devices, particularly in supercapacitors and batteries^49,50^.

Materials with the designation MOF-76(Ln) (Ln = lanthanide(III)) and chemical composition [Ln(BTC)(H_2_O)] representing a group of homotypic porous lanthanide MOFs were first published in 2005 by Rosi et al.^17^. Their crystal structure consists of Ln(III) cations, which, through *syn-syn* and *syn-anti* coordination of BTC(-III) carboxylate groups (same as HKUST-1), form 1D helical chains (see Figs. 1d, e). The described coordination of building components leads to the formation of a 3D framework with 1D sinusoidal channels of size 7 × 7 Å^2^, while the coordinated water molecules are oriented inside the channels, as depicted in Fig. 1(f). The uniqueness of these compounds lies in the transformability and high thermal stability of the framework (up to 600 °C in the air atmosphere). To date, several compounds containing lanthanide(III) ions such as Ce^51–56^, Nd^57,58^, Sm^58,59^, Eu^58–63^, Gd^58,64–66,109,111^, Tb^17,58–60,67–77^, Dy^59,78,79^, Ho^58,80^, Er^58^, Tm^80^, Yb^58,81,82^, Lu^51,83^ and Y^68,82,84,85^ were prepared and investigated in different application areas. These materials were tested as gas adsorbents for CO_2_^51,54,65,66,78,80,84^, CH_4_^65,80^, H_2_^57,77,78,109^, and NH_3_^82^. They have also been studied as heterogeneous catalysts in reactions such as cyanosylilation^58^, Knoevenagel condensation^51^, esterification^81^, synthesis of NH_3_^53^, and oxidative desulfurisation reactions^67^. Because of the specific fluorescence and luminescence properties of Ln(III) ions within MOF-76, these compounds find interesting applications as sensors for sensing cations and anions^56,71,73,76^, aromatic pollutants^59^, drugs^60,61,70^, pesticides^75^, and small organic molecules^62,69,74^. MOF-76(Ln) were also studied as humidity sensors, showing an excellent response even at low moisture values^57,77,109^. Other fields of applications are magnetism and magnetic refrigerators^63–65,79^, electrochemical hydrogen and oxygen production^55^, photocatalytic degradation of ammonia^68^ or additives in LiS batteries^111^.

In recent times, the preparation of MOF-containing composite materials has become an important research topic. MOFs can be incorporated into composite structures to enhance their mechanical, thermal, electrical, and functional properties^86–88^. MOFs with small dimensions (ideally nanometers) are needed to prepare composite materials. Since MOF materials are crystalline compounds with sizes ranging from microns to millimetres, it is necessary to reduce their particle size. Several methods can be employed:

*Solvothermal/solvent-assisted methods* This involves the synthesis of MOFs using solvents under high-temperature and high-pressure conditions. By carefully controlling the reaction parameters, such as molar ratio, *pH*, reaction time and temperature, it is possible to obtain nano-sized MOF particles^89^. The mentioned procedure was used for the direct synthesis of nano-MOFs such as MIL-88A (100–200 nm)^90^, MOF-5 (100–150 nm)^91^, ZIF-8 (40–50 nm)^92^ or MIL-89 (20–40 nm)^93^.

*Modulators* are used in the direct synthesis of MOF materials, influencing the size and morphology of the particles due to the competitive interaction between the modulator/linker and the central atom. As an example of a modulator, acetic acid can be listed, the amount of which during synthesis can affect the size of the resulting [Cu_2_(NDC)_2_(dabco)]_n_ particles from 5 to 80 nm^94^. A frequently used modulator in the preparation of Zr(IV) or Hf(IV) MOFs is benzoic or formic acid, the amount of which affects the grain size of UiO-66 (20–1000 nm)^95^.

*Microemulsion methods* This synthesis effectively yields uniform and unimodal nanoscale MOF crystals, while the principle lies in the formation of a microemulsion of two immiscible liquids in the presence of surfactants or emulsifiers. The resulting nanodroplets containing a solution of reactants serve as a nanoreactor in which the desired MOF is produced. The mentioned method was used in the synthesis of „nano “ HKUST-1 (30–140 nm)^96^, Fe-soc-MOF (100 nm)^97^ and MOF-5 (80 nm)^98^.

*Sonication* Ultrasonic waves are applied to disperse and break down MOF aggregates, leading to the reduction of particle size, or the energy is sufficient for a chemical reaction to take place, leading to the formation of MOFs. Sonication can be done in solvents or solvent mixtures to achieve better dispersion and smaller particle sizes. The mentioned procedure was used for downsizing or synthesis of nanoMOFs, for example, TMU-69 (100–700 nm)^99^ and MOF-2 (200–300 nm)^100^.

*Template-assisted methods* Using a sacrificial template with specific pore dimensions, MOF synthesis can be guided to yield reduced-sized particles. The template could be subsequently removed, leaving behind smaller-sized MOF particles. Examples of templates/substrates include MOF materials themselves (e.g. ZIF-8/MOF-5, MIL-101/ZIF-8, UiO-66-NH_2_/IRMOF-3^101^) or mesoporous oxides (e.g. MOF-5/SBA-15 (silica)^102^, UiO-66/zirconia^103^) with sufficiently large pores in which another MOF material can be grown.

*Grinding/milling* MOF crystals can be mechanically ground or milled to reduce their particle size. This method involves using a mortar and pestle, ball mill, or other grinding equipment to break down larger crystals into smaller particles. The mentioned technique was used for downsizing as-synthesised Gd-pDBI with dimensions of 0.5 mm to 140 nm^104^ or ZIF-8 (100 nm)^105^. As another example, MIL-53 was milled for various time intervals (2, 4, 6 and 8 min) and then applied as an acetylene adsorbent. However, the authors do not report the specific particle size^106^ after mechanical activation. Mechanical milling can be efficiently used as a synthesis tool to reach MOFs. Processing materials in high-energy ball mills leads to the process of mechanical activation, which, apart from bringing down the particle size and specific surface area increase, also brings about other effects such as defects introduction and microstrain, which might be attractive for particular applications^107^.

It is important to note that the choice of particle size reduction method depends on the specific MOF material and the desired particle size range. Each method has its advantages and limitations, and optimisation is required to achieve the desired particle size while maintaining MOF stability and properties. While many studies focus on the synthesis of MOFs, reports on their mechanical downsizing are rare and herein lies the uniqueness of the present study.

The objective of the presented work was to preparare two MOFs containing a trimesate linker, HKUST-1 and MOF-76, and reduce their grain size through mechanical activation using (a) a high-energy ball mill and by (b) a hand grinding with a mortar and pestle. HKUST-1, one of the first MOF materials prepared, has significantly attracted the interest of the scientific community. As introduced above, it is widely studied in various application areas, making it suitable candidate for this study. MOF-76 has high thermal stability reaching 600 °C^51,80^, which is advantageous durig high-energy ball milling when the temperature increases. The effect of the selected milling conditions was assessed via a statistical Taguchi orthogonal array approach. The crystallite size was determined using dry particle size measurement via laser diffraction, dispersion in methanol using the DLS method (wet way), and SEM. The stability and characterisation of the materials before and after mechanical treatment were studied using IR, PXRD and nitrogen adsorption/desorption measurements. Subsequently, the materials were studied as adsorbents of carbon dioxide at 0 °C and pressure up to 100 kPa.

## Materials and methods

### Used chemicals

Copper(II) nitrate trihydrate (Cu(NO_3_)_2_·3H_2_O, 99%, Sigma Aldrich), gadolinium nitrate hexahydrate (Gd(NO_3_)_3_·6H_2_O, 99.9%, Sigma Aldrich), benzene-1,3,5-tricarboxylic acid (H_3_BTC, 95%, Sigma Aldrich), *N,N´*-dimethylformamide (DMF, 99.8%, ACS reagent) and ethanol (EtOH, 99.5%, Sigma Aldrich)) were used for synthesis of HKUST-1 and MOF-76 compounds.

### Synthesis of MOF materials

The MOF materials HKUST-1 and MOF-76 were synthesised according to the procedures described in^108,109^ and^110,111^, respectively.

*HKUST-1* 2.00 g (9.52 mmol) of benzene-1,3,5-tricarboxylic (trimesic) acid and 4.00 g (16.56 mmol) of copper(II) nitrate trihydrate were dissolved in 90 cm^3^ of the mixed solvents DMF/EtOH/H_2_O (1:1:1 / v:v:v) in a glass 200 cm^3^ autoclave. After dissolving the reactants, the reaction mixture was heated to 85 °C with a heating rate of 0.5 °C min^-1^ for 12 h and then cooled to room temperature with a cooling rate of 1 °C min^-1^. HKUST-1 in the form of blue cubic crystals was isolated by filtration under reduced pressure, washed with ethanol, and dried in a stream of air (yield 2.386 g, 56% based on trimesic acid). Elemental analysis for HKUST-1: Cu_3_C_24_H_36_O_22_N_2_ ([Cu_3_(BTC)_2_(H_2_O)_3_]⋅2DMF⋅5H_2_O; 895.18 g mol^-1^): clcd. C 32.20%, H 4.05%, N 3.13% found: C 32.36%, H 4.11%, N 3.08%.

*MOF-76* 0.40 g (1.90 mmol) of benzene-1,3,5-tricarboxylic (trimesic) acid and 2.00 g (4.43 mmol) of gadolinium(III) nitrate hexahydrate were dissolved in 170 cm^3^ of the mixed solvents DMF/EtOH/H_2_O (6:5:5/v:v:v) in a glass 200 cm^3^ autoclave. After dissolving the reactants, the reaction mixture was heated to 80 °C with a heating rate of 0.5 °C min^-1^ for 12 h and then cooled to room temperature with a cooling rate of 1 °C min^-1^. MOF-76 in the form of colourless needle-shaped crystals was filtered off, washed with ethanol, and dried in a stream of air (yield 0.712 g, 82% based on trimesic acid). Elemental analysis for MOF-76: Gd_1_C_12_H_12_O_8_N_1_ ([Gd(BTC)(H_2_O)]⋅DMF; 455.48 g mol^-1^): clcd. C 31.64%, H 2.66%, N 3.08% found: C 31.71%, H 2.63%, N 3.12%;

### Ball milling and hand grinding procedures

*Ball milling procedure* The MOFs were mechanically activated in a Pulverisette 7 premium line planetary ball mill (Fritsch Germany) using a 20 mL achate milling chamber and air atmosphere. The selected conditions of mechanical activation were optimised using the Design of Experiment methodology, namely the orthogonal 3^3^ array designed via the Taguchi method^112^. This allowed us to find the optimum parameters much faster (instead of all 27 experiments, which are theoretically possible to investigate all the combinations of studied parameters, just nine needed to be performed). Namely, the rotation speed of the planet carrier, milling time and balls diameter (three factors) were changed on three levels (details are provided in Table 1 in the Results and Discussion). The experiments were planned according to Taguchi's design using the Minitab14 software (Minitabl Ltd., UK).

**Table 1:** Conditions (T1-T9) used in the mechanical activation of MOF materials designed via 3^3^ Taguchi orthogonal array and the *d*_*50*_ values obtained in a dry way for HKUST-1 and MOF-76 samples treated under given conditions.

	Rotation speed [rpm]	Milling time [min]	Balls [number x size]	*d*_*50*_ (µm) for HKUST-1	*d*_*50*_ (µm) for MOF-76
orig	–	–	–	18.28	8.21
T1	250	5	50 × 5 mm	11.41	0.86
T2	250	15	3 × 10 mm + 25 × 5 mm	22.84	0.70
T3	250	30	6 × 10 mm	24.375	0.70
T4	375	5	3 × 10 mm + 25 × 5 mm	21.20	0.72
T5	375	15	6 × 10 mm	19.61	0.78
T6	375	30	50 × 5 mm	73.85	0.69
T7	500	5	6 × 10 mm	23.00	0.82
T8	500	15	50 × 5 mm	64.22	0.73
T9	500	30	3 × 10 mm + 25 × 5 mm	112.80	0.78

*Hand grinding procedure* A portion of the original HKUST-1 and MOF-76 samples were dispersed in methanol and hand grinded occasionally every working day for 20 days in the mortar and pestle. The reproducibility of hand grinding process was carried out by three different persons with different physical and body proportions: researcher 1 (man, 190 cm, 100 kg), researcher 2 (man, 185 cm, 80 kg) and researcher 3 (woman, 170 cm, 55 kg). The hand grinding of HKUST-1 was performed for seven days (20 min each day) and prepared materials were designated as researcher 1, 2 and 3.

### Characterisation

The elemental analysis (carbon, hydrogen and nitrogen content) of HKUST-1 and MOF-76 was performed using the elemental analyser Vario MICRO from Elementar Analysensysteme GmbH.

Infrared (IR) spectroscopy was used to study the bond types in organic components of MOF materials and phase changes. The infrared spectra of samples were measured on FT-IR spectrometer Avatar 6700 in the wavenumber range of 400–4000 cm^-1^ using the ATR (attenuated total reflectance) technique with 64 repetitions for a single spectrum and resolution of 4 cm^-1^.

Powder X-ray diffraction experiments (PXRD) were performed to study phase changes and stability of MOF materials before and after the mechanical activation. The measurements were carried out on a Rigaku Ultima IV multipurpose diffractometer using CuK*α* radiation (*λ* = 1.54056 Å) in the reflection geometry. Diffracted photons were recorded using a NaI scintillation detector in the 2 theta range of 5–60° with a measurement speed of 0.5° min^-1^.

Mastersizer 2000E with dry feeder Scirocco 2000 M (dry air transport medium) was used for particle size measurement. Each measurement starts with the calibration of the laser and the optical system. After optical alignment, a blank measurement was taken immediately before the test sample measurement, and a particle-free transport medium (dry air) was used under the same instrument conditions (air pressure) to be employed for the test sample measurement. These background signals were subtracted later from the detector signals coming from the measurement of the material of interest. The density of the powder samples was measured with a helium pycnometer system (AccuPyc II 1340). Calibration was performed using a calibration standard by running a protocol, the result of which was stored as a standard, and the measurement of experimental samples was started, according to ASTM B923.

Another method of particle size measurement was chosen using the DLS technique for particles dispersed in a methanol solution (wet way). The samples were measured on a Zetasizer Nano ZS (Malvern Panalytical, UK). This measurement was aimed at measuring the size of small particles present in the ground sample. A solution of 100 mg dm^-3^ in 96% ethanol was prepared. Before measurement, each sample was ultrasonicated (USD) for 1 min and then measured at several time intervals (0, 10, 30 min; 0 min = immediate analysis) so that the larger particles sank to the bottom and the size of the smaller particles was measured. The technique uses a He–Ne laser with a wavelength of 632.8 nm as the radiation source and backscatter detection at an angle of 173°.

The other method used to study the particle size and morphology of untreated and selected milled materials was field-emission scanning electron microscopy (FE-SEM). The measurements were performed on a TESCAN A MIRA3 instrument (TESCAN, Brno, Czech Republic). The grains of the materials were deposited on an adhesive carbon tape attached to a holder, which was subsequently inserted into the instrument, and the measurements were carried out.

Nitrogen physisorption analysis was conducted to assess the porous characteristics of the original, hand grinded, and ball milled samples, following the procedure outlined in^108,113^. Prior to performing the physisorption experiments, the samples were activated at the temperature of 100 °C (HKUST-1) or 300 °C (MOF-76) for 20 h in a fine vacuum. So-called pressure-controlled heating was used to repress a possible elutriation of the powdered samples in the sample cell due to fast vapour desorption from the samples' porous structure. The adsorption isotherms were recorded over a relative pressure range of approximately 10^–4^–0.995 *p/p*_*0*_ (relative pressure). The void volume of a sample cell containing a sample was determined by a helium-free NOVA® approach. The N_2_ adsorption isotherms were used to calculate the BET area (*S*_*BET*_), respecting Rouquerol's method^114^. The volume of micropores (*V*_*micro*_, pore diameter < 2 nm) and mesopores (*V*_*meso*_, pore diameter 2–50 nm) was calculated from the pore size distribution (PSD) curves. These were obtained by fitting the experimental adsorption data by a Non-Local Density Functional Theory (NLDFT) adsorption kernel (ASiQwin software, Quantachrome Instruments), assuming a cylindrical pore shape for silica.

Carbon dioxide adsorption isotherms were measured at 0 °C up to ca. 100 kPa as described in^108,113^. The same activation procedures and the NOVA® technique for assessing the void volume, as used for nitrogen adsorption at − 196 °C, were applied. The CO_2_ sorption capacity of the samples was represented by the quantity of adsorbed CO_2_ at 100 kPa (in cm^3^_STP_ g^-1^, mmol g^-1^, wt. %).

## Results and discussion

### Particle size determination of ball milled and hand grinded samples

One of the most straightforward approaches to observing the effect of mechanical activation on the treated material is the investigation of its particle size. In Table 1, the *d*_*50*_ (median particle size) values for the original and ball milled samples are provided.

The particles of the original HKUST-1 were almost two times coarser than the MOF-76 ones. The obtained results clearly show the opposite effect of milling on the particle size of the two investigated MOFs. In the case of HKUST-1, the particle size reduction was achieved only upon the mildest conditions used (T1), while a very significant reduction in the submicron range was achieved in the case of MOF-76. However, the effect of individual parameters in the latter case seems to be insignificant, as the results for all MOF-76-T(1–9) samples are very similar.

**Figure 2:**
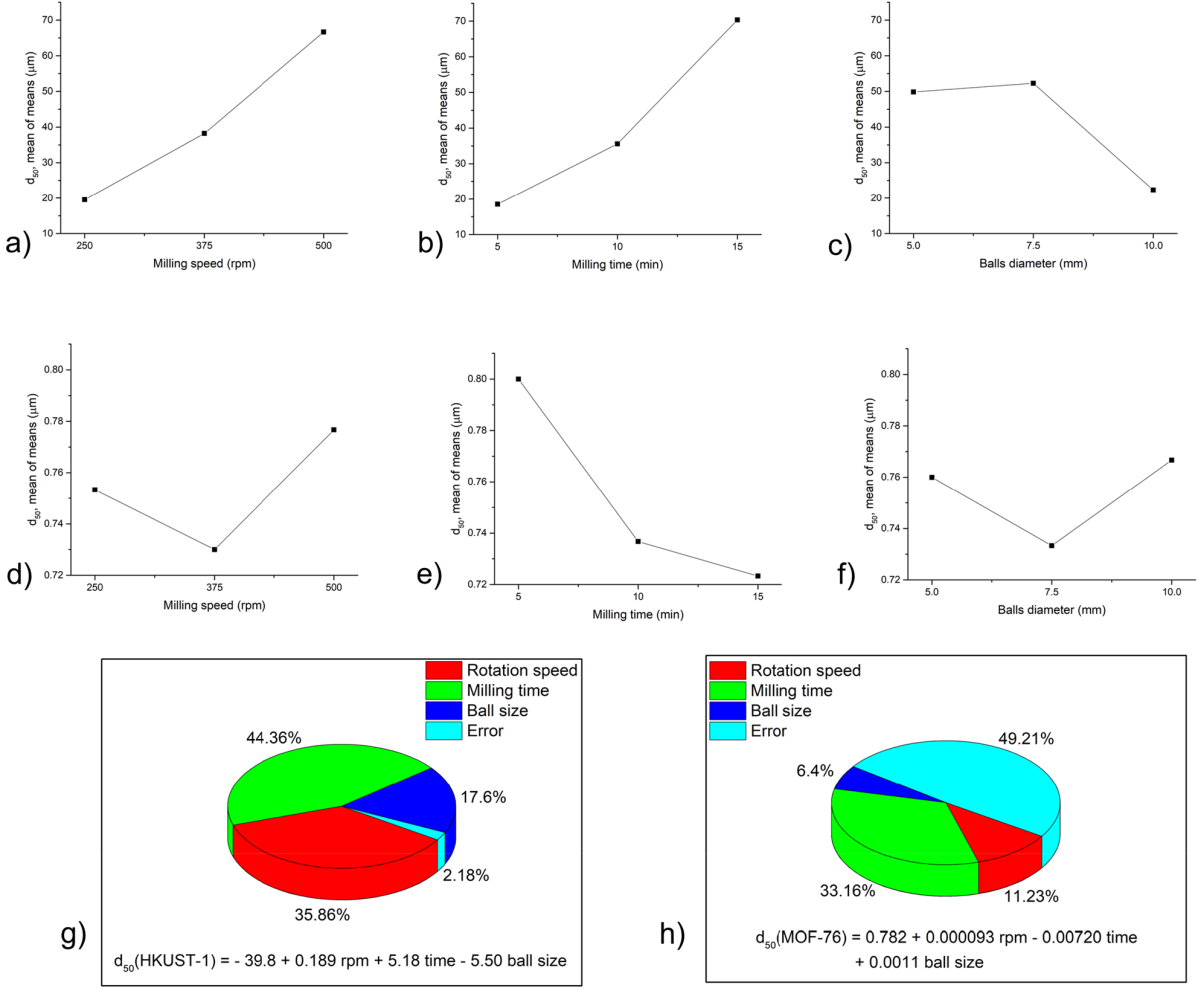
Result of Taguchi calculations for mean values of *d*_*50*_ for HKUST-1: (**a**–**c**) and MOF-76 (**d**–**f**) regarding: (**a**, **d**) milling speed; (**b**, **e**) milling time and (**c**, **f**) balls diameter (in this case the diameter 7.5 mm stands for the combination of 5 mm and 10 mm balls). The contribution of individual parameters on the *d*_*50*_ value for g) HKUST-1 and h) MOF-76 was calculated by one-way Analysis of Variance Analysis (ANOVA). The corresponding regressions are provided.

Taguchi calculations were performed to determine the effect of various milling conditions on the particle size of MOFs. As an output, *d*_*50*_ values (i.e. median particle sizes for each sample) were used. The smaller-the-better approach was used for calculations, i.e. the aim was to obtain the lowest particle size possible. The results from calculations for HKUST-1 and MOF-76 are summarised in Fig. 2.

The optimum parameters for the mechanical activation of MOFs are the ones where the minimum particle size was reached. This accounts for 250 rpm, 5 min and 10 mm balls for HKUST-1 and 375 rpm, 15 min and 7.5 mm balls (i.e., a combination of 10 mm and 5 mm balls) for MOF-76. However, in the latter case, the change in particle size is very small (the *d*_*50*_ values are in the range between 0.7 and 0.9 µm), thus all the used conditions yield very similar results in terms of particle size.

In order to investigate the contribution of individual parameters to the reduction of particle size, ANOVA analysis was used, and regressions describing the relationship between input parameters and the output for both MOFs were calculated (see Fig. 2).

The results show significantly different results for the two MOFs. For HKUST-1, the ANOVA seems to be quite reliable, as the error in calculation was only slightly above 2%. The most important parameter is the milling time, followed by rotation speed, and the lowest impact was detected for ball size. On the other hand, for MOF-76, the error reaches almost 50%, which is most probably the result of very similar *d*_*50*_ values for these samples, as discussed above. Nevertheless, milling time also seems to be the most important factor here. The contribution of rotation speed, albeit still the second most important, is much lower than for HKUST-1, and the impact of ball size is almost negligible. A much lower influence of the milling parameters for MOF-76 is also reflected in the calculated regressions, where the coefficients are at least three orders of magnitude lower than in the case of HKUST-1.

DLS measurements provide information on the (hydrodynamic) diameter of the particles, which may differ from dry particle size measurements due to the more accurate analysis of small particles that the mechanical routes of material disintegration can achieve. These small particles may be important for various applications, such as for the synthesis of composite materials.

**Figure 3:**
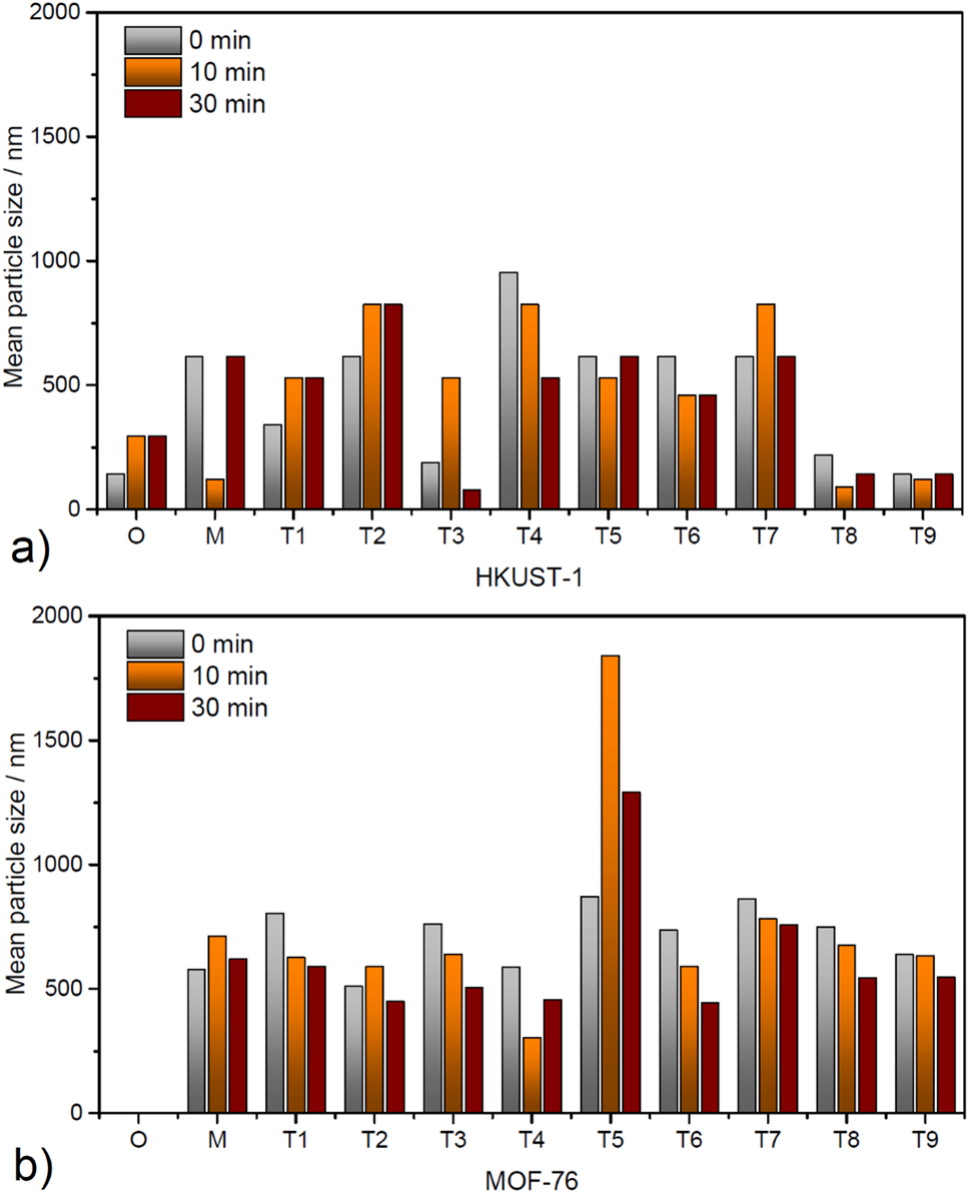
Mean particle size in wet state determined by DLS measurements of (**a**) HKUST-1 and (**b**) MOF-76 methanol suspensions immediately after ultrasound sonification (0 min), 10 min, and 30 min after sonification containing original (O) material, hand grinded (M) sample and compounds after ball milling procedures under condition T1-9.

Given that the presence of micrometric particles together with nanometric particles can compromise the reliability of nanometric particle analysis, the experiment was designed as follows. The samples were dispersed in methanol, placed in an ultrasonic bath for 1 min before measurement, immediately measured (0 min), and then again after 10 and 30 min. During that time, the suspensions were left freely stand without any manipulation. During this time, the larger micrometric particles were sedimented, and it was possible to measure the smaller nanometric particles present in the suspension. The average particle size of HKUST-1 and MOF-76 materials determined by DLS measurements at different sedimentation times (0, 10 and 30 min) are summarised in Fig. 3 and Figs S2, 3 in ESI.

For the original HKUST-1 (O) sample (see Fig. S2 in ESI) after ultrasound treatment, the particle size was 142 nm, but with a prolonged time, the particles clustered, and within 10 min, the particle size stabilised at around 300 nm. It can be seen that after 30 min, the larger-sized particles have dropped to the bottom, and the particles that remained in suspension have a relatively narrow width of distribution.

For the hand grinded (M) HKUST-1, the particle sizes measured at time 0 and 30 min were approximately identical. This can be explained by the fact that immediately after the removal of the suspension from the ultrasonication, the particles are separated. After 10 min, some of the particles aggregate, forming larger clusters that sediment and are no longer visible in subsequent measurements. At this time we can see particles around 120 nm, which were not detected at time 0 min due to the presence of larger particles. After 30 min, particles of 300 nm are again detected, which seems to be a stable particle size for this material (M) HKUST-1. For the HKUST-1 samples after ball milling under conditions T1—T9, the particle sizes ranging from 80 to 825 nm were observed. The results can be divided into two groups. For samples T3, T8 and T9, sizes below 200 nm were achieved after 30 min—larger particles settled to the bottom of the measuring cell with time, and the smallest particle size values were achieved here. For samples T1, T2, T4, T5, T6 and T7, the particle size values after 30 min range from 400 to 800 nm. Summing up, for the HKUST-1 sample, ball milling under conditions T3, T8 and T9 was able to achieve significantly smaller particle size than the as-synthesised sample.

The particle size of the original MOF-76 (O) could not be determined by DLS, as the material crystallises in the form of needles with a size of several millimetres.

For the MOF-76 hand grinded (M) sample (see Fig. S3 in ESI), it can be observed that the measured mode particle size does not change with time, while the mean increases due to higher polydispersity and broader particle size distribution. In this case, most of the particles are in the size of ca. 600 nm, and with increasing time, the grains agglomerate. As a result, the detected particle size increases.

For MOF-76 samples after ball milling under conditions T1—T9, no significant trend in particle size shift can be observed either by changing the ball milling parameters or by measuring the same sample at 0, 10 and 30 min after its 1-min ultrasonication. The typical size of the prepared particles ranges from 400 to 600 nm. This size is certainly smaller than the untreated MOF-76 (O) sample, which the DLS technique could not measure because its grain size was larger than the detectable particle size of the technique. Alternatively, large agglomerates of particles appeared in the sample, reflecting the incident laser beam and obscuring the smaller, detectable particles. When compared to the hand grinded (M) sample, the samples processed through ball milling demonstrated particle sizes that were either smaller or equivalent.

### Scanning *electron* microscopy

**Figure 4:**
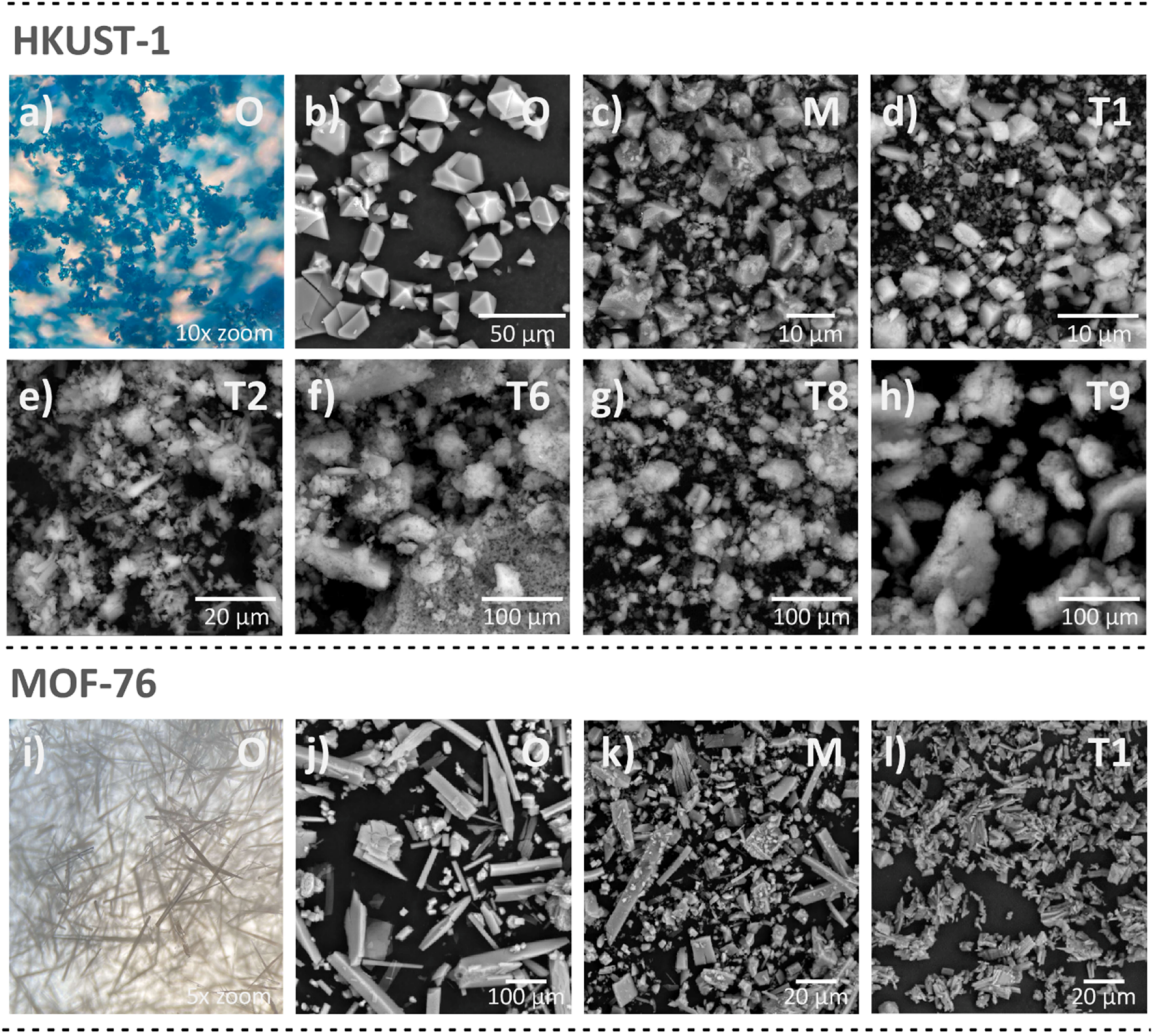
(**a**) Optical (10 × zoom) and (**b**) SEM image of the original HKUST-1 (O). SEM images with different magnification for (**c**) hand grinded (M) and (**d**) T1, (**e**) T2, (**f**) T6, (**g**) T7 and (**h**) T9 ball milled HKUST-1 materials. (**i**) Optical (5 × zoom) and (**j**) SEM image of the original MOF-76 (O). SEM images with different magnification for(**j**) hand grinded (M) and (**d**) T1 ball milled MOF-76 samples.

Figure 4 shows the optical images created by microscope and SEM images of the original compounds HKUST-1 and MOF-76, samples after hand grinding and selected ball milled materials. Since the particle size of the ball milled HKUST-1 materials varied based on the median particle size values (*d*_*50*_, see Table 1) in the range of 10–120 μm, the representatives of different sizes (T1 ~ 10 μm; T2 ~ 25 μm; T6 ~ 75 μm; T8 ~ 65 μm and T9 ~ 110 μm) were selected. Since the demonstrated particle size for MOF-76 showed approximately the same values regardless of milling conditions, only one representative (T1 ~ 0.9 μm) was chosen.

As can be seen from Figs. 4 (a,b) and (i,j), the mechanically untreated materials have the shape of octahedra, or long needles for HKUST-1 and MOF-76, respectively. The crystal size of HKUST-1 ranges from a few micrometers to up to 30 μm, and MOF-76 forms long needle-shaped crystals with a size of a few millimeters. In the case of hand-milled materials (M), there is a reduction in grain size to a level of up to 10 μm and deformation of the octahedral shape for HKUST-1 (see Fig. 4c), and a shortening of the needle-shaped crystals, forming cube- or block- shaped particles for MOF-76 (see Fig. 4k). Ball milling HKUST-1 samples exhibit different particle size and shape depending on the milling conditions (see Figs. 4d–h). After mechanical treatment, the original octahedral shape is destructed, and the particle size is reduced, but they agglomerate to form larger aggregates that exceed the size of the original HKUST-1. It can be concluded that the particle size correlates with the *d*_*50*_ values (see Table 1 and the scale bar in Figs. 4d–h for comparison). For the ball milled MOF-76 material (T1, see Fig. 4l), a more pronounced reduction in particle size can be observed compared to the original or hand grinded sample.

### Infrared spectroscopy

All prepared materials were characterised by FTIR spectroscopy (see Fig. S1 in ESI). A series of infrared spectra of HKUST-1 and MOF-76 materials and their derived forms obtained by different types of ball milling and hand grinding were analysed. The infrared spectra of HKUST-1 exhibit similarity with the non-treated material (O) only in the case of hand grinded material (red curve in Fig. S1a in ESI), depicting the asymmetric vibrations of carboxylate groups at wavenumbers of 1645 and 1585 cm^−1^, as well as symmetric vibrations at 1445 and 1373 cm^−1^. Ball milling leads to changes in the area of COO^—^vibrations, which can be attributed to a change in the structure of the given materials. The structural changes were also confirmed by PXRD patterns (see subsection below). Fig. S1b displays the IR spectrum of the original, hand grinded, and ball milled MOF-76 substances. Predominantly featured are bands corresponding to organic linker vibrations. Specifically, for benzene-1,3,5-tricarboxylate, the asymmetric vibration of the carboxylate anion *ν*_*as*_(COO^−^) at 1610 cm^−1^ and its symmetric vibration *ν*_*s*_(COO^−^) at 1371 cm^−1^ are evident in all milled materials. The presence of the carboxylate group is further supported by the deformation vibration *δ*(COO^−^) at 711 cm^−1^. Additionally, a band at 1542 cm^−1^ signifies the aromatic valence vibration *ν*(CH)_ar_ of the BTC aromatic scaffold. According to infrared spectroscopy of MOF-76 hand/ball milled material, structural changes did not occur, which was also confirmed by PXRD analysis.

### Powder X-ray diffraction

**Figure 5:**
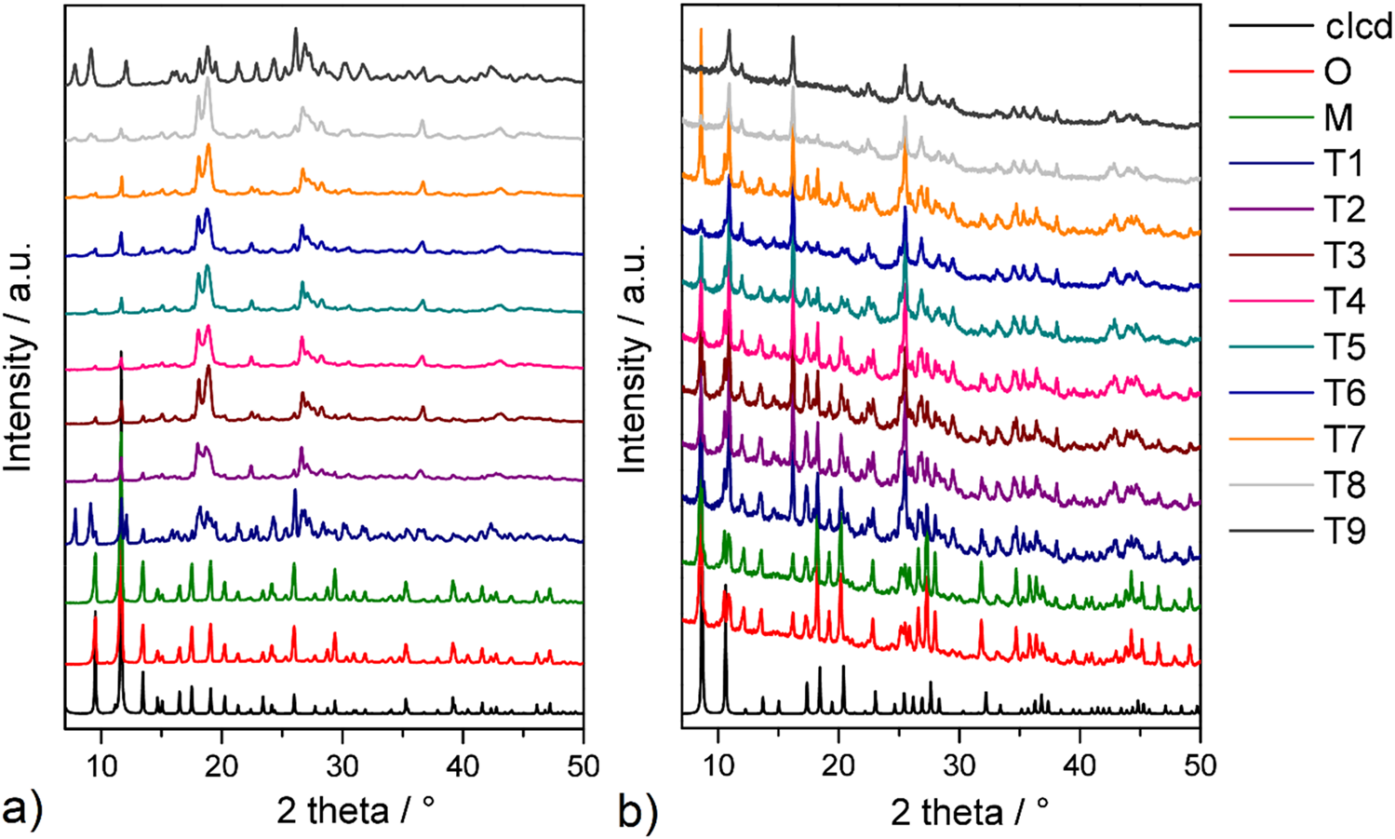
PXRD patterns of simulated (clcd), original (O), hand grinded (M) and ball milled materials under different conditions (T1-T9) of (**a**) HKUST-1 and (**b**) MOF-76.

All prepared materials were studied using powder X-ray diffraction (PXRD) analysis in the range of 2*θ* angles from 7 to 35° (see Fig. 5). Figure 5(a) contains the PXRD patterns of the milled samples compared to the pristine HKUST-1 material. A good agreement was observed between the calculated PXRD pattern from single crystal X-ray data, the original material, and the hand grinded sample. The main diffraction peaks at 2*θ* values of 8.6°, 10.6°, 12.3°, 13.7°, 15.0° and 16.2° corresponding to the (220), (222), (400), (311), (420) and (422) crystallographic planes, respectively, and are the same as those reported for the PXRD patterns of HKUST-1^115^. Ball milling of HKUST-1 under T1-T9 conditions led to a decreasing and exterminating of bands, which was caused by the effect of grinding, and thus changes in the crystal structure especially in connection with partial or complete amorfization or its collapse (see Fig. 5a)^116–118^. Otherwise, for the metal–organic framework MOF-76, a good agreement in the PXRD patterns between the calculated, hand grinded and ball milled materials (T1-T9) was observed. In materials of type T6 and T8-T9, a decrease in diffraction intensities was observed, most probably due to the decreasing crystallite size (see Fig. 5b).

To provide a rationale for why MOF-76 is more stable than HKUST-1 upon ball milling, it is necessary to consider several structural and chemical factors that contribute to the stability of MOFs under mechanical treatment. In terms of structural stability, it is necessary to consider metal-cluster geometry and connectivity. MOF-76 forms highly connected, robust metal-oxo 1D chains with connectivity 7 or higher, leading to a more rigid and stable framework. HKUST-1 contains paddle-wheel units with connectivity 5 in its framework. This structure is comparatively less connected and can be more prone to deformation and breaking of metal–ligand bonds under mechanical stress. Linker strength and flexibility also influence the resulting stability of MOFs. Since the MOF-76 and HKUST-1 compounds contain the same linker, this consideration is eliminated. Another structural factor is the bond strength between the central atom (Gd(III)/Cu(II)) and the donor oxygen atom on the carboxylate group of the BTC(-III) linker. In terms of bond energy, Gd-O is stronger since gadolinium has a higher charge (oxidation state) than copper. By applying the HSAB theory^119^, the higher stability of MOF-76 can also be pointed out. Since oxygen (carboxylate group) is a hard base, it prefers to form more stable compounds with hard acid. Lanthanides (Ln(III)) are generally classified as hard acids because of their low electronegativity, medium ionic radius and high oxidation state. Cu(II), on the other hand, is classified as an intermediate acid. Since the hard base encourages the formation of a more stable complex with the hard acid, MOF-76 is thermodynamically more stable than HKUST-1. The above reasons are also reflected in the thermal stability of the materials, which extends to 600 °C for MOF-76^51,80^ and 320 °C for HKUST-1^108^. It is known that in the milling process, there is an increase in temperature when the balls collide with the reactor walls. As MOF-76 is more thermostable compared to HKUST-1, this is reflected in its higher stability after ball milling.

### Nitrogen adsorption

**Figure 6:**
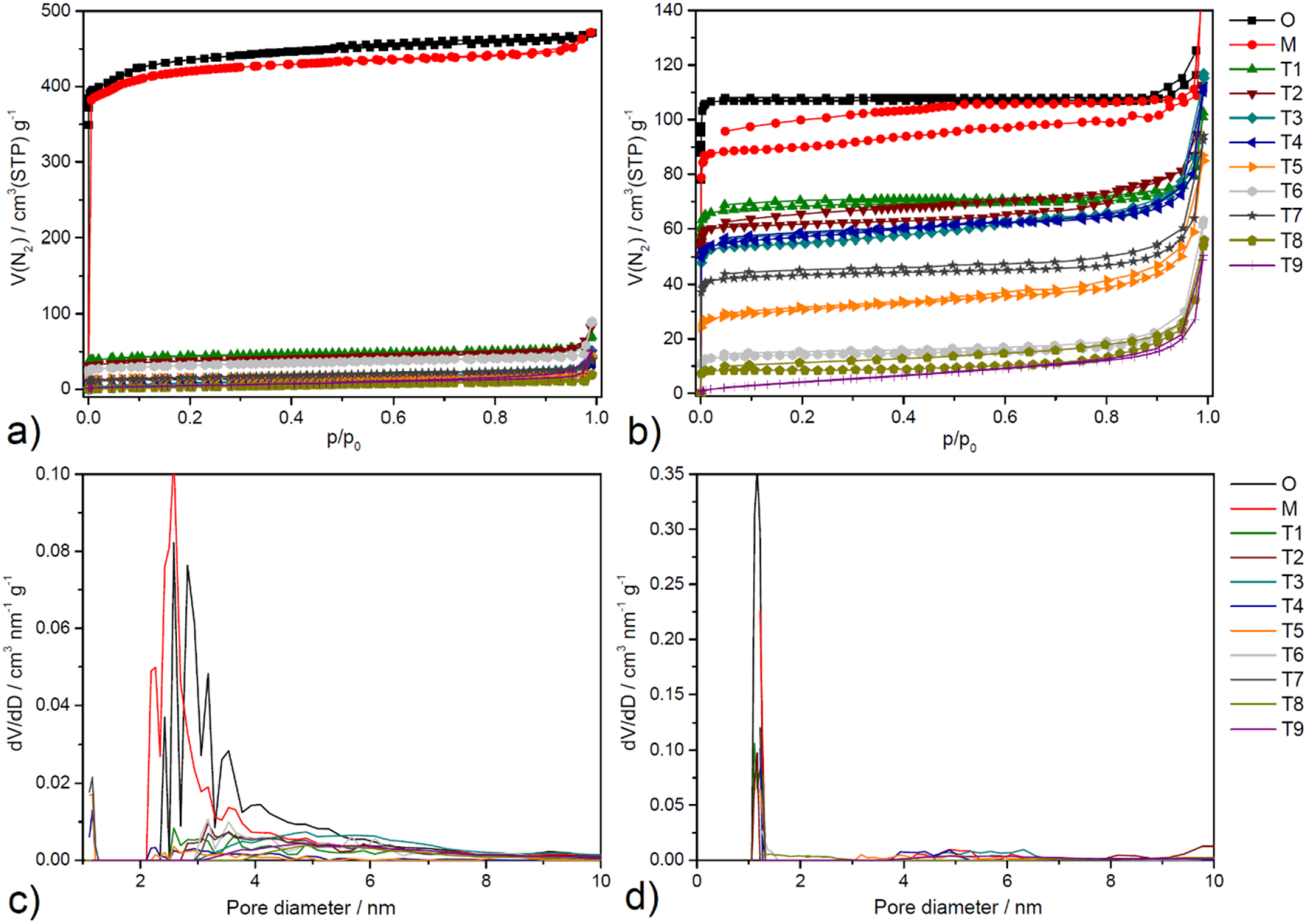
Nitrogen adsorption/desorption isotherms at -196 °C of original (O), hand grinded (M) and ball milled samples (T1-T9) of a) HKUST-1 after activation at 100 °C and c) MOF-76 after activation at 300 °C. The pore size distribution curves for c) HKUST-1 and d) MOF-74 were calculated by NLDFT.

Porous properties of the original, hand grinded, and ball milled samples were analysed by the gas physisorption. Nitrogen adsorption/desorption isotherms measured at -196 °C are illustrated in Fig. 6(a) and (b) for HKUST-1 and MOF-76, respectively.

The isotherms for the original HKUST-1 and MOF-76 are of the type *Ia/Ib* according to the latest IUPAC classification^114^, suggesting a predominantly microporous structure for these materials. This is in line with the results provided in Table 2, where micropore volume (*V*_*micro*_) significantly exceeds mesopore volume (*V*_*meso*_) for each MOF material. Pore size distribution curves indicate that the mesopores in HKUST-1 range from 2.2 to 8 nm, with the most common pore size around 3 nm (Fig. 6c). In contrast, MOF-76 is characterised by an absence of mesoporosity, as shown in Table 2, possessing exclusively microporous characteristics with micropores measuring 1.2 nm (Fig. 6d). Considering all provided porous parameters, HKUST-1 has a micropore volume fourfold larger than that of MOF-76, thus being more porous of the two MOF materials.

**Table 2:** Calculated textural parameters (micropore/mesopore pore volume, BET area) of prepared materials from nitrogen adsorption/desorption data obtained at -196 °C.

	HKUST-1			MOF-76		
	*V*_*micro*_ [cm^3^ g^-1^]	*V*_*meso*_ [cm^3^ g^-1^]	*S*_*BET*_ [m^2^ g^-1^]	*V*_*micro*_ [cm^3^ g^-1^]	*V*_*meso*_ [cm^3^ g^-1^]	*S*_*BET*_ [m^2^ g^-1^]
O	0.677	0.088	1712	0.170	0.000	463
M	0.611	0.112	1659	0.139	0.028	380
T1	0.064	0.028	170	0.111	0.020	282
T2	0.047	0.044	142	0.097	0.046	258
T3	0.000	0.041	33	0.081	0.053	227
T4	0.017	0.011	50	0.085	0.049	231
T5	0.021	0.008	57	0.039	0.057	117
T6	0.040	0.030	117	0.019	0.033	56
T7	0.014	0.029	54	0.065	0.037	178
T8	0.000	0.026	16	0.006	0.044	37
T9	0.000	0.035	21	0.000	0.040	19

For the hand grinded HKUST-1, the volume of micropores decreased slightly from 0.677 to 0.611 cm^3^ g^-1^ (10% decrease). On the other hand, their mesopore volume increased by 27% (from 0.088 to 0.112 cm^3^ g^-1^), as seen in Fig. 6 and Table 2. The porosity of MOF-76 proved to be less resistant to hand grinding, with an 18% decrease in micropore volume observed (from 0.170 to 0.139 cm^3^ g^-1^), while the mesopore volume increased from 0 to 0.028 cm^3^ g^-1^. The increase in mesoporosity alongside decrease in microporosity suggests a transition where micropores are widened into mesopores. This is probably due to partial disruption of the crystalline structure, where milling and shaking introduce defects into the material's microporous framework^120–122^.

It is evident that the ball milling procedure significantly reduced the porosity of both MOF materials (Fig. 6 and Table 2). Under relatively mild conditions (T1), the micropore volume was reduced to 0.064 cm^3^ g^-1^ for HKUST-1 and 0.111 cm^3^ g^-1^ for MOF-76. However, conditions that are too severe (T9) can lead to a complete loss of the microporous structure. The nitrogen adsorption isotherms for all ball milled samples exhibit increased uptake at relative pressures greater than 0.95 *p/p*_*0*_. This observation is associated with the creation of large mesopores and/or small macropores, which is attributed to the introduction of defects (as discussed above) into the framework of the MOF structure during the ball milling process.

Prior to examining the outcomes of the Taguchi method and ANOVA analysis related to the BET area (*S*_*BET*_), it is important to highlight that even under mild ball milling conditions, the reduction in microporosity is significantly more pronounced compared to that observed in hand grinded samples. The decrease in microporosity for hand grinded samples is only partial, with micropores being converted into smaller mesopores. This transformation may hold particular interest for the development of (partially) mesoporous MOF materials, which have potential use in various applications^123^.

Taguchi's approach has also been used to evaluate the effect of various ball milling parameters on the BET area (*S*_*BET*_) value of both MOFs. We are, of course, aware that the BET method is not valid for the microporous adsorbents, but we needed to have a numerical value to be used as an input for Taguchi calculations, so the S_*BET*_ value seems to be a good selection as an estimation. Already, the first glimpse shows that the BET area was significantly reduced for all T1-T9 samples in both cases, the effect being much more pronounced in the case of HKUST-1. This is in accordance with the results of particle size analysis, where *d*_*50*_ values for HKUST-1 were, in most cases, higher than the original material, which, in general, means an agglomeration process.

**Figure 7:**
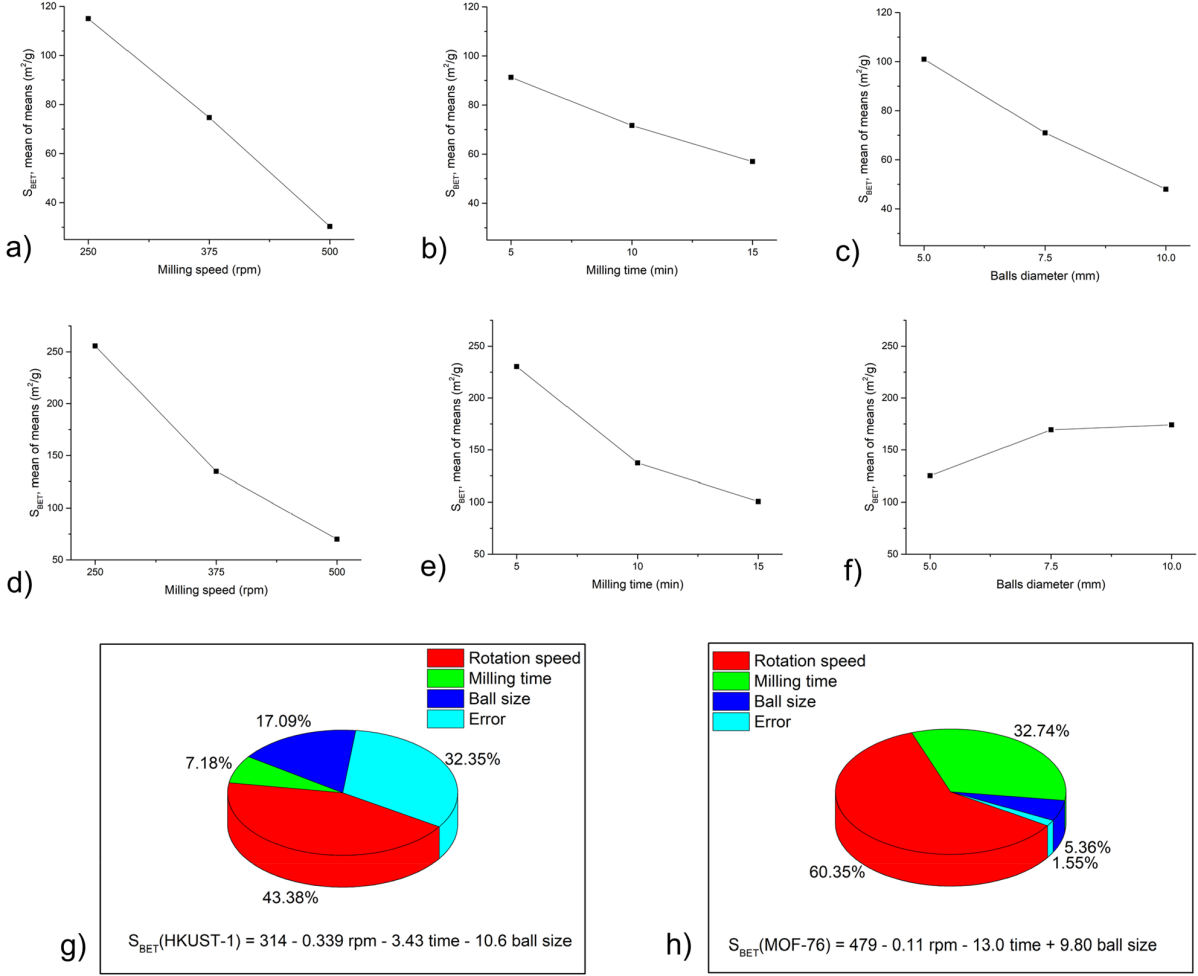
Result of Taguchi calculations for mean values of *S*_*BET*_ for HKUST-1 (**a**–**c**) and MOF-76 (**d**–**f**) regarding: (**a**, **d**) rotation speed; (**b**, **e**) milling time, and (**c**, **f**) balls diameter (in this case the diameter 7.5 mm stands for the combination of 5 mm and 10 mm balls). The contribution of individual parameters on the *S*_*BET*_ value for (**g**) HKUST-1 and (**h**) MOF-76 was calculated by one-way Analysis of Variance Analysis (ANOVA). The corresponding regressions are provided.

In the case of *S*_*BET*_ Taguchi calculations, the larger-the-better approach was used, i.e. the aim was to achieve the highest *S*_*BET*_ value possible. The results are provided in Fig. 7(a) and (b) below. Because the *S*_*BET*_ value was significantly reduced due to milling, the mildest milling conditions turned out to be optimal ones (i.e. rotation speed 250 rpm and milling time 5 min). This was valid for both MOFs. However, the difference was discovered in the influence of ball diameter. Whereas in HKUST-1, the smallest balls were the most suitable, the largest ones were most useful in the case of MOF-76. This underlines the different responses of the two MOFs under study to the milling treatment.

The ANOVA analysis (see Figs. 7a–h) has shown that for both MOFs, the rotation speed was the most impactful parameter influencing the *S*_*BET*_ value, being more important for MOF-76 than for HKUST-1. The second most important parameter was ball size for HKUST-1, whereas it was milling time for MOF-76. Interestingly, the results were vice versa in terms of the error in comparison with *d*_*50*_ ANOVA analysis (see Fig. 2g and h). Namely, the error was much higher in the case of HKUST-1, whereas for MOF-76, it was negligible. The regressions clearly show the negative influence of milling on the *S*_*BET*_ value, as all the coefficients are negative (with the exception of + 9.80 in front of ball size for MOF-76).

### Carbon dioxide sorption

**Figure 8:**
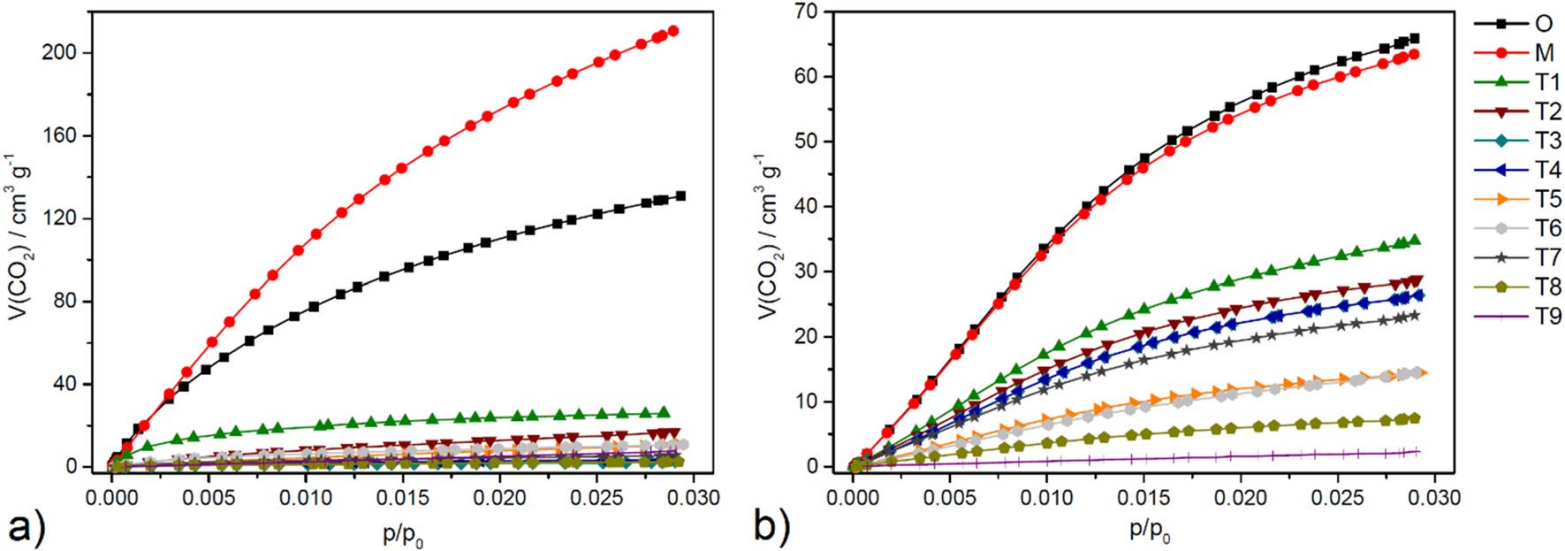
Carbon dioxide adsorption isotherms at 0 °C of original (O), hand grinded (M) and ball milled samples (T1-T9) of a) HKUST-1 after activation at 100 °C and c) MOF-76 after activation at 300 °C.

Carbon dioxide adsorption isotherms for original (O), hand grinded (M), and ball milled (T1-T9) samples of HKUST-1 and MOF-76 are depicted in Fig. 8(a) and (b), respectively, and their adsorption capacities are summarised in Table 3. It is observed that the CO_2_ adsorption capacity for HKUST-1 can significantly decrease from 25.70 wt. % to as low as 0.50 wt. % under the T8 ball milling condition. The mildest ball milling condition (T1) results in a reduction to 5.13 wt. %, which constitutes a fivefold decrease. MOF-76 exhibits greater resistance against the reduction in CO_2_ adsorption capacity due to ball milling, evidenced by a twofold decrease in the capacity observed (from 12.93 wt. % to 6.82 wt. % for T1). It is to be noted that the CO_2_ adsorption capacity of MOF-76 materials ranges from 11 to 13 wt. % at 0 °C and 1 bar, and depends on the central atom present in the framework and the activation temperature of the compound^51,54,65,80^. Under the most severe ball milling conditions (T9), the CO_2_ adsorption capacity can be diminished to 0.45 wt. %. The reductions in CO_2_ adsorption capacity can be attributed to the decrease in micropore volume, as CO_2_ is preferentially adsorbed in these micropores under the specified measurement conditions^124–128^.

**Table 3:** CO_2_ adsorption capacities at 0 °C and 100 kPa in different units (cm^3^ g^-1^, mmol g^-1^, wt. %) of prepared samples.

	CO_2_ adsorption capacity						
	HKUST-1				MOF-76		
	[cm^3^_STP_ g^-1^]	[mmol g^-1^]	[wt. %]		[cm^3^_STP_ g^-1^]	[mmol g^-1^]	[wt. %]
O	130.87	5.84	25.70		65.87	2.94	12.93
M	211.71	9.40	41.37		63.62	2.84	12.49
T1	26.12	1.17	5.13		34.73	1.55	6.82
T2	17.25	0.77	3.39		28.76	1.28	5.65
T3	2.56	0.11	0.50		26.39	1.18	5.18
T4	3.60	0.16	0.71		26.39	1.18	5.18
T5	10.92	0.48	2.14		14.47	0.65	2.84
T6	10.82	0.48	2.12		14.47	0.65	2.84
T7	5.75	0.26	1.13		23.31	1.04	4.58
T8	2.56	0.11	0.50		7.43	0.33	1.46
T9	7.81	0.35	1.53		2.30	0.10	0.45

In the following text, ball milled samples will be discussed in a framework of the Taguchi methodology and ANOVA analysis.

**Figure 9:**
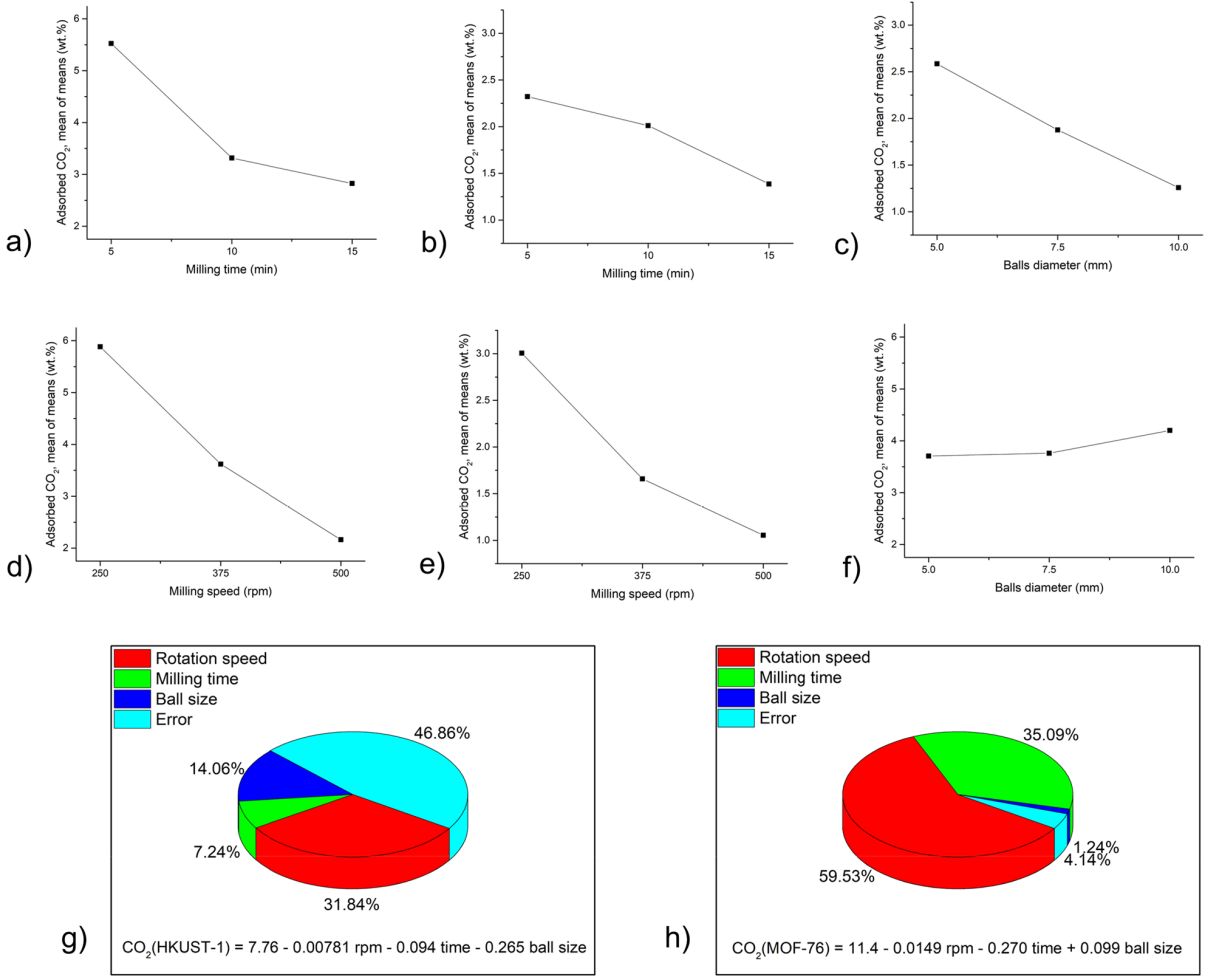
Result of Taguchi calculations for mean values of adsorbed CO_2_ for (**a**–**c**) HKUST-1 and MOF-76 (**b**–**f**) regarding: (**a**, **d**) rotation speed; (**b**, **e**) milling time (top right) and (**c**, **f**) balls diameter (in this case the diameter 7.5 mm stands for the combination of 5 mm and 10 mm balls). The contribution of individual parameters on the amount of adsorbed CO_2_ value for (**g**) HKUST-1 and h) MOF-76 was calculated by one-way Analysis of Variance Analysis (ANOVA). The corresponding regressions are provided.

Taguchi methodology has also been used to evaluate the effect of various ball milling parameters on CO_2_ adsorption by both MOFs. The results more-or-less mirror the effects detected for *S*_*BET*_ values, i.e. reduction in both MOFs, being much more significant for HKUST-1. Similarly to *S*_*BET*_ Taguchi calculations, the larger-the-better approach was also used for CO_2_ adsorption. The results are provided in Fig. 9(a–h) below.

The obtained results with regard to the optimum milling conditions are absolutely the same as those detected for *S*_*BET*_ calculations, including the peculiarity of the larger ball size being beneficial for MOF-76. Also, the results of the ANOVA analysis (Fig. 9h) are very similar, with rotation speed being the most important parameter and a large error in the case of HKUST-1. Also, the coefficients in the regressions are negative, with the exception of the ball size influence of MOF-76.

Samples subjected to ball milling exhibited no enhancement in CO_2_ adsorption capabilities, which is crucial for capture, storage, and utilisation applications. In contrast, hand grinding procedures did not significantly deteriorate or even have a positive effect on CO_2_ adsorption of MOF-76 and HKUST-1, respectively. Specifically, the hand grinded MOF-76 sample preserved its CO_2_ adsorption efficiency at 12.49 wt. %, closely aligning with the 12.93 wt.% observed in the original MOF-76. Notably, the CO_2_ sorption capacity of the HKUST-1 increased from 25.70 wt. % (O) to 41.37 wt. % after hand grinding (M), marking a 38% enhancement. It should be noted that the adsorption capacity of hand grinded HKUST-1 (41.37 wt.%) is higher than for amine-modified HKUST-1 materials treated with ethylenediamine (22.31 wt.%)^50^, ammonia (31.8 wt.%)^129^ or diethylenetriamine (33.09 wt. %)^50^ under the same experimental conditions (0 °C and 1 bar). This increase suggests the additional porosity made by hand grinding is readily accessible to CO_2_ molecules at a relatively high measurement temperature of 0 °C. In contrast, nitrogen molecules, despite their similar kinetic radius, could not access these pores because their adsorption occurred only at the cryogenic temperature of − 196 °C. This phenomenon is attributed to the well-documented diffusion restrictions at cryogenic temperatures^114,124^. As a result, the hand grinded (M) sample displayed somewhat lower porosity properties (see Table 2) compared to the original (O) sample. It is speculated that prolonged hand grinding, may lead to the exposure of previously closed or inaccessible pores within the HKUST-1 framework, consequently enhancing its CO_2_ sorption capacity significantly.

To validate our hypothesis, we determined the micropore volume from the CO_2_ adsorption isotherms by fitting them with the Grand Canonical Monte Carlo (GCMC) adsorption kernel for both the original and hand grinded HKUST-1, which yielded micropore volumes of 0.423 cm^3^ g^−1^ and 0.745 cm^3^ g^−1^, respectively (results not tabulated). The micropore volume for the hand grinded sample is 22% larger than that obtained from nitrogen adsorption at − 196 °C. Moreover, while the micropore volume assessed by N_2_ at − 196 °C encompasses the entire range of micropores (up to 2 nm), the volume calculated from CO_2_ adsorption at 0 °C is limited to pores narrower than 1.47 nm. Hence, it can be inferred that the total micropore volume might be equal to or even exceed that measured by nitrogen adsorption. We can conclude that the gentle hand grinding of HKUST-1 can enhance its CO_2_ adsorption capacity by unlocking previously closed or inaccessible pores, despite nitrogen adsorption at − 196 °C suggesting a reduction in porosity. Hand grinding of MOF materials has shown the potential for increasing CO_2_ adsorption capacities. This method is obviously not suitable for applications due to its laboriousness. It is possible that under certain conditions, such as utilising a small amount of liquid additives or utilising different mills (e.g. mixer mill), also a ball milling could lead to such a positive outcome. Nevertheless, our findings highlight the importance of careful handling of MOF materials to improve their adsorption or other properties.

### Reproducibility of hand grinding

**Figure 10:**
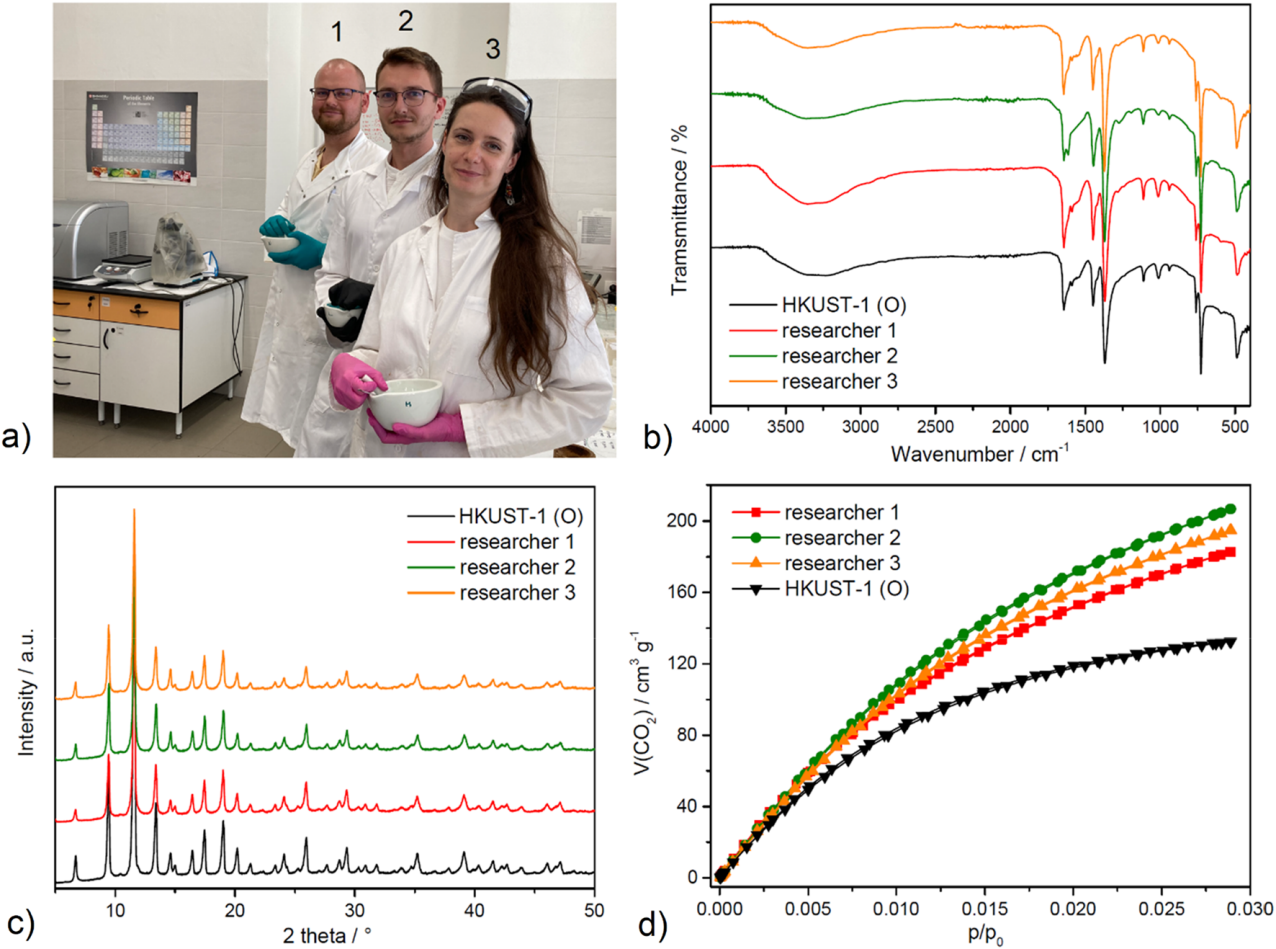
(**a**) Researchers who carried out manual milling with corresponding numbering. Results of (**b**) IR, (**c**) PXRD and CO_2_ adsorption isotherms measured at 0 °C for the freshly prepared original HKUST-1 and its three hand grinded forms (researcher 1–3).

Since the process of hand milling may be subjective and depending on the person realizing the grinding, the question of results reproducibility and the resulting adsorption properties arises. To answer the question of manual milling reproducibility, an experiment was designed in which three different persons with different physical and body proportions performed hand milling of HKUST-1 for one week (20 min each day, see Fig. 10a). The pristine HKUST-1 and the resulting ground samples prepared by the three different researchers (designated as researcher 1, 2 or 3) were characterized by infrared spectroscopy (IR), powder X-ray diffraction analysis (PXRD) and carbon dioxide adsorption at 0 °C, the results of which are summarized in Fig. 10 and Table 4. As is evident, the materials retained their structure after hand milling, as the IR spectra (Fig. 10b) and PXRD patterns (Fig. 10c) of the milled materials matched those of the mechanically untreated sample. The results of carbon dioxide measurements at 0 °C and 100 kPa (see Fig. 10d) showed adsorbed volumes of 132.50, 182.77, 195, and 206.78 cm^3^_STP_ g^-1^ for newly prepared original HKUST-1, researcher 1, 3, and 2, respectively. Consequently, the average adsorbed volume was 195 ± 12 cm^3^ STP g^-1^ fro researcher 1, 2 and 3, corresponding to an acceptable deviation of only 6%. This confirms that the hand grinding procedure is well-reproduced.

**Table 4:** CO_2_ adsorption capacities at 0 °C and 100 kPa in different units (cm^3^ g^-1^, mmol g^-1^, wt. %) of newly synthesized original HKUST-1 and hand milled samples prepared by reasearchr 1, 2 and 3.

	CO_2_ adsorption capacity		
	[cm^3^_STP_ g^-1^]	[mmol g^-1^]	[wt. %]
HKUST-1 (O)	132.50	5.91	26.02
Researcher 1	182.77	8.15	35.89
Researcher 2	194.82	8.69	38.25
Researcher 3	206.78	9.23	40.60

## Conclusion

The investigation aimed to analyse the effects of mechanical activation, particularly through ball milling and hand grinding, on the properties of two metal–organic frameworks (MOFs), HKUST-1 and MOF-76, containing trimesate linker. The study precisely examined changes in particle size, structural characteristics, porosity, and carbon dioxide adsorption capacities resulting from different milling conditions, the effect of which, in the case of ball milling, was assessed via a statistical method- Taguchi orthogonal array.

The results revealed distinct responses of HKUST-1 and MOF-76 to mechanical activation. HKUST-1 demonstrated a significant reduction in particle size and an alteration in crystal structure under specific ball milling conditions, leading to changes in porosity and ultimately impacting CO_2_ adsorption capacity. Conversely, MOF-76 exhibited less pronounced changes in particle size and structural integrity upon milling, maintaining relatively stable porosity and CO_2_ adsorption capabilities compared to HKUST-1. Ball milling might also be suitable, provided that careful consideration is given to selecting the appropriate conditions to prevent material degradation. Hand grinding has emerged as a gentle method to at least preserve the CO_2_ adsorption capacity of the original MOF (MOF-76) or even increase it by 38% in the case of HKUST-1. This suggests the possibility of opening previously inaccessible pores within the MOF framework through gentle hand grinding treatment, thereby improving CO_2_ sorption efficiency. In the case of HKUST-1 and MOF-76 materials, hand grinding was found to be well-reproduced, making it the preferred method over ball milling.

Furthermore, Taguchi methodology and ANOVA analysis provided insights into the influence of milling parameters on particle size, surface area, and CO_2_ adsorption. These analyses highlighted the importance of milling time, rotation speed, and ball size in determining the outcome of mechanical activation, with different MOFs showing varying sensitivity to these parameters.

In conclusion, the study underscores the complex interplay between milling parameters, structural changes, porosity, and adsorption properties of MOFs. It highlights the potential of mechanical activation as a versatile tool for tailoring the properties of MOF materials for various applications, particularly in gas adsorption and separation processes. Moreover, the findings emphasise the importance of meticulous handling and optimisation of milling conditions to achieve desired material properties, opening routes for further research in the field of MOF synthesis and engineering.

### Supplementary Information

Supplementary Figures.

## Data Availability

The datasets used and/or analysed during the current study available from the corresponding author on reasonable request.
