# Supplementary material for: The influence of HKUST-1 and MOF-76 hand grinding/mechanical activation on stability, particle size, textural properties and carbon dioxide sorption
Source: Sci Rep. 2024 Jul 4;14:15386. doi: 10.1038/s41598-024-66432-z (PMC11224341; doi:10.1038/s41598-024-66432-z)
Supplement: Supplementary file 1 — Supplementary Figures. [file 41598_2024_66432_MOESM1_ESM.pdf]

SUPPLEMENTARY MATERIAL

The influence of HKUST-1 and MOF-76 hand grinding/mechanical activation on stability, particle size, textural properties and carbon dioxide sorption

Tomáš Zelenka[a+], Matej Baláž[b], Marta Férová[a], Pavel Diko[c], Jozef Bednarčík[c], Alexandra Királyová[d], Ľuboš Zauška[d], Radovan Bureš[e], Pooja Sharda[f], Nikolas Király[d], Aleš Badač[a], Jana Vyhlídalová[a], Milica Želinská[d], Miroslav Almáši[d*]

[a] Department of Chemistry, Faculty of Science, University of Ostrava, 30. Dubna 22, CZ-702 00 Ostrava, Czech Republic

[b] Institute of Geotechnics, Slovak Academy of Sciences, Watsonova 45, SK-040 01 Košice, Slovak Republic

[c] Institute of Experimental Physics, Slovak Academy of Sciences, Watsonova 47, SK-040 01 Košice, Slovak Republic

[d] Department of Inorganic Chemistry, Faculty of Science, P. J. Šafárik University, Moyzesova 11, SK-041 01 Košice, Slovak Republic

[e] Institute of Materials Research, Slovak Academy of Sciences, Watsonova 47, SK-040 01 Košice, Slovak Republic

[f] Department of Physics, School of Applied Science, Suresh Gyan Vihar University, I-302017 Jaipur, India

[+]tomas.zelenka@osu.cz; *miroslav.almasi@upjs.sk

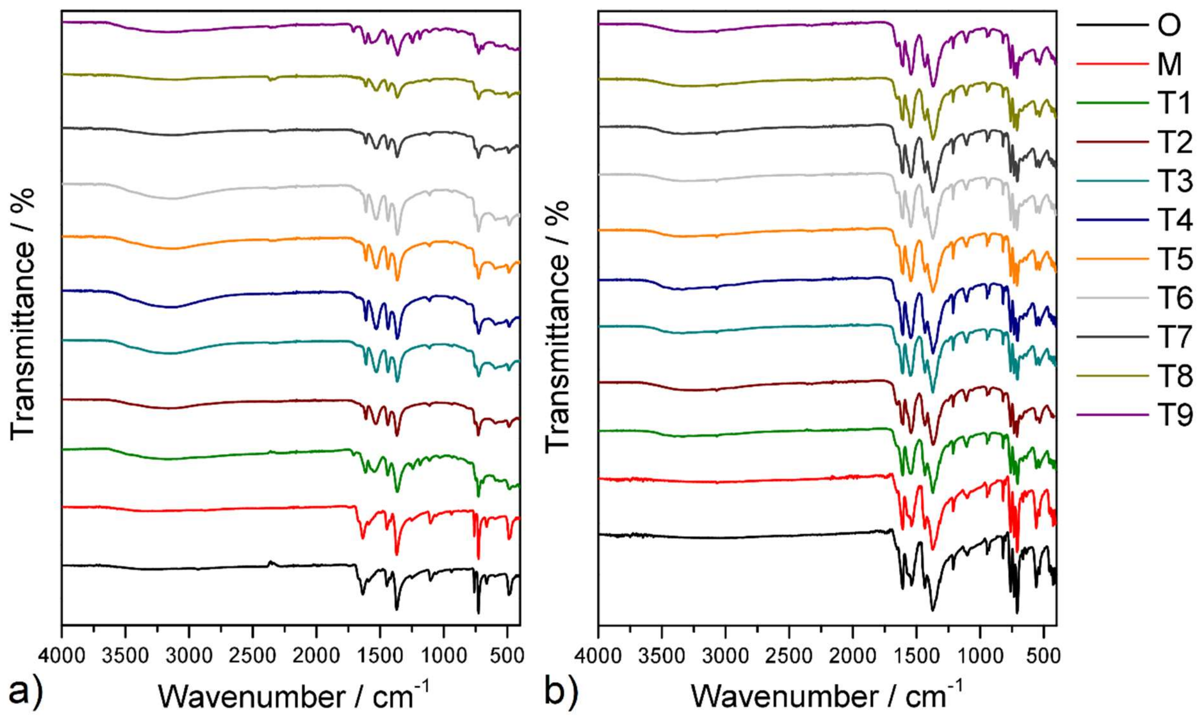

Figure S1 Infrared spectra of original (O), hand grinded (M) and ball milled materials under different grinding conditions (T1-T9) of a) HKUST-1 and b) MOF-76.

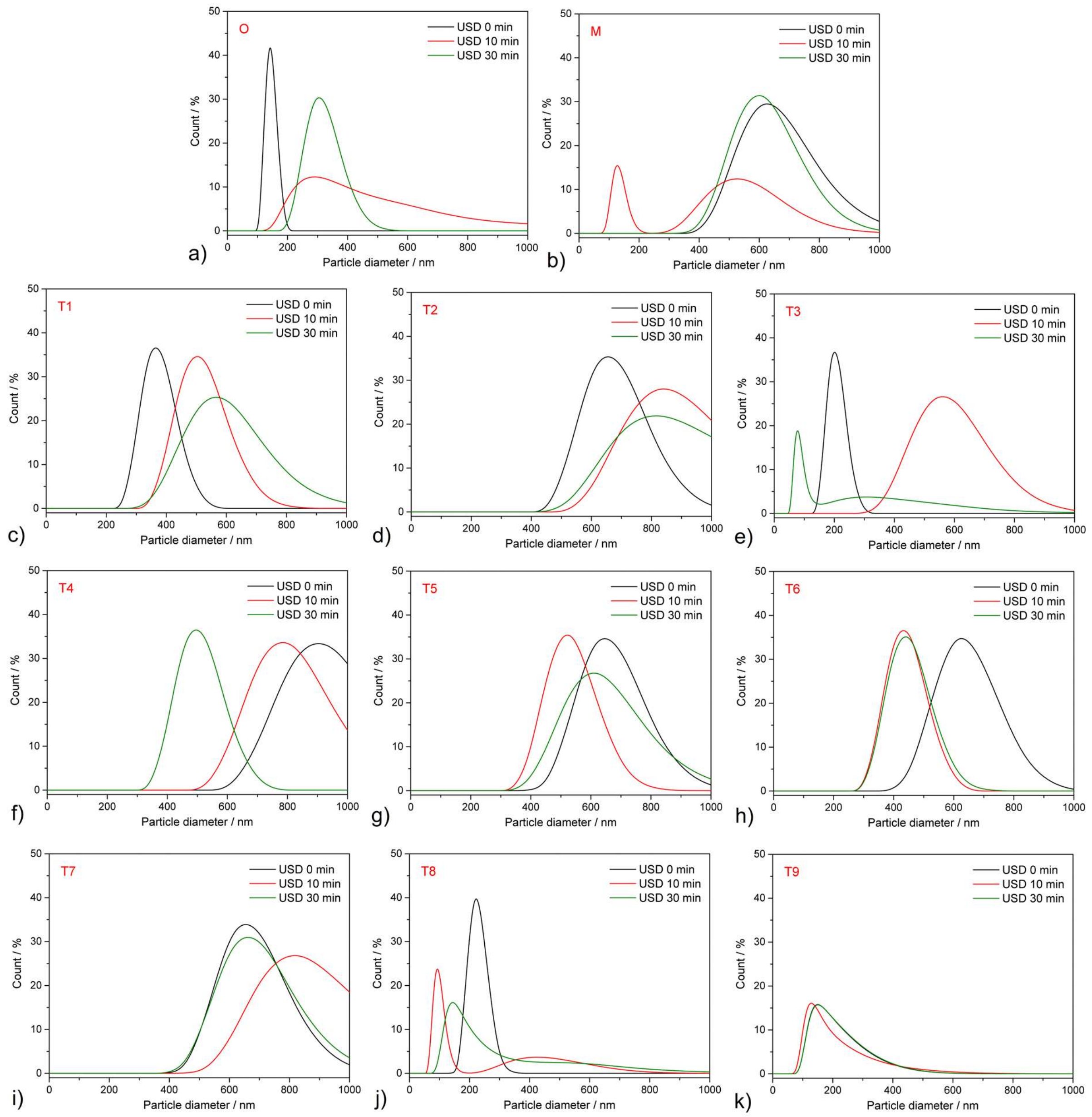

Figure S2 Occurrence and particle diameter determined by DLS measurements of HKUST-1 methanol suspensions immediately after ultrasound sonification (USD 0 min), 10 minutes (USD 10 min), and 20 minutes after sonification (USD 20 min) containing a) original (O) material, b) hand grinded (M) sample and compounds after ball milling under condition c) T1, d) T2, e) T3, f) T4, g) T5, h) T6, i) T7, j) T8 and k) T9.

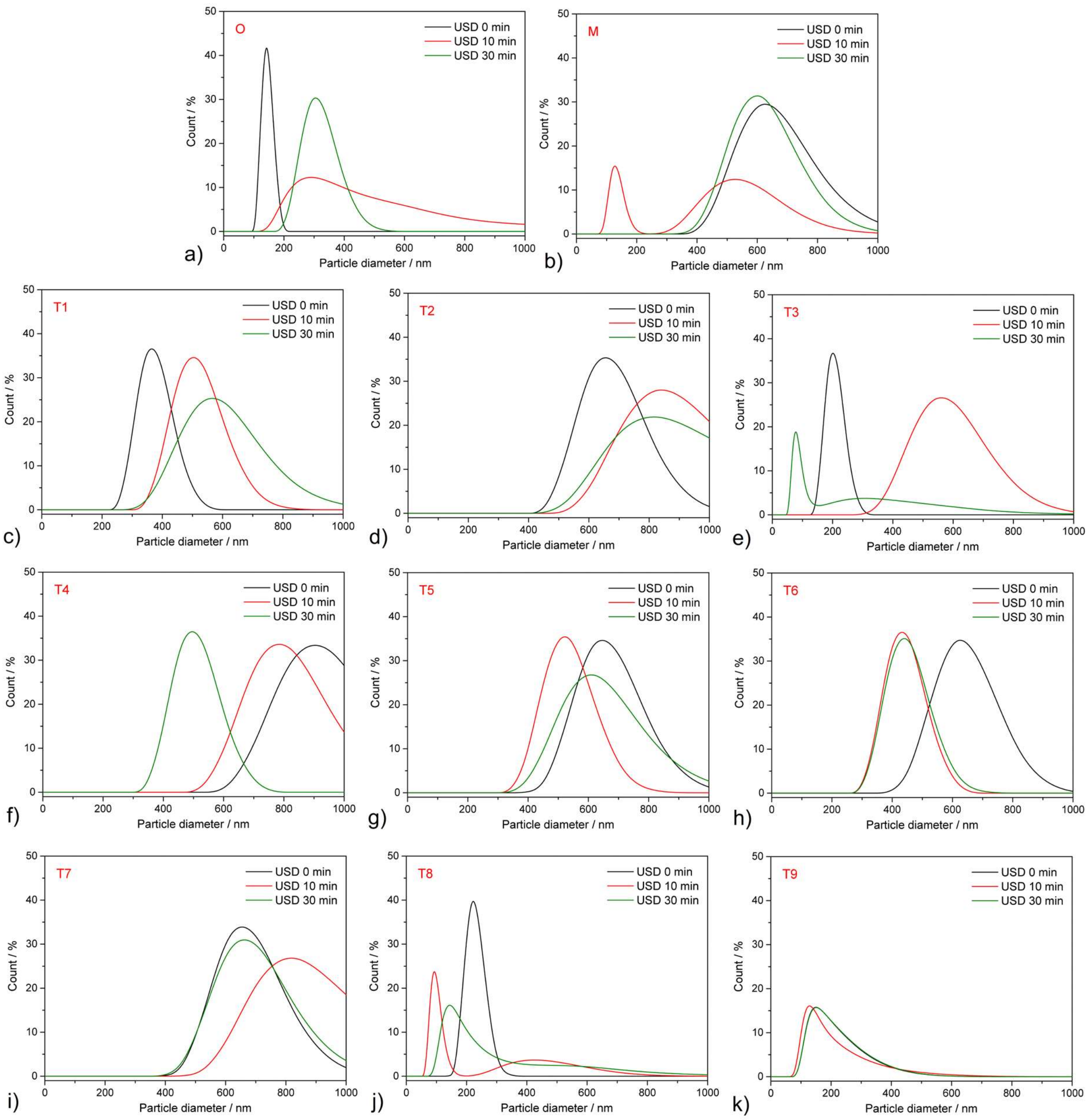

Figure S3 Occurrence and particle diameter determined by DLS measurements of MOF-76 methanol suspensions immediately after ultrasound sonification (USD 0 min), 10 minutes (USD 10 min), and 20 minutes after sonification (USD 20 min) containing a) original (O) material, b) hand grinded (M) sample and compounds after mechanochemical ball milling under condition c) T1, d) T2, e) T3, f) T4, g) T5, h) T6, i) T7, j) T8 and k) T9.
